# The impact of electroacupuncture on anxiety-like behavior and gut microbiome in a mouse model of chronic restraint stress

**DOI:** 10.3389/fnbeh.2023.1292835

**Published:** 2023-11-16

**Authors:** Jie Bai, Jia-Quan Wei, Qian Tian, Fen Xue, Wen Zhang, Hong He

**Affiliations:** Department of Psychiatry, Xi'an Gaoxin Hospital, Xi'an, China

**Keywords:** electroacupuncture, chronic restraint stress, gut microbiota, anxiety, species

## Abstract

**Introduction:**

Electroacupuncture (EA) is a beneficial physiotherapy approach for addressing neuropsychiatric disorders. Nevertheless, the impact of EA on the gut microbiome in relation to anxiety disorders remains poorly understood.

**Methods:**

To address this gap, we conducted a study using a chronic restraint stress (CRS) mouse model to investigate the anti-anxiety outcome of EA and its influence on gut microbiota. Our research involved behavioral tests and comprehensive sequencing of full-length 16S rRNA microbiomes.

**Results:**

Our findings revealed that CRS led to significant anxiety-like behaviors and an imbalance in the gut microbiota. Specifically, we identified 13 species that exhibited changes associated with anxiety-like behaviors. Furthermore, EA partially alleviated both behaviors related to anxiety and the dysbiosis induced by CRS.

**Discussion:**

In summary, this study sheds light on the alterations in gut microbiota species resulting from CRS treatment and brings new light into the connection between EA’s anti-anxiety effects and the gut microbiota.

## 1. Introduction

Anxiety disorders manifest with both mental and bodily symptoms, such as anxiety, fear, nervousness, lack of air, and dizziness. They affect approximately 12.1% of the global population (Charlson et al., 2019). These disorders rank ninth among health-related reasons for disability and contribute to 3.3% of the global disease burden (Vos et al., 2017). The primary treatments for people with anxiety currently encompass pharmacotherapy and psychotherapy. Serotonin-norepinephrine reuptake inhibitors (SNRIs) and selective serotonin reuptake inhibitors (SSRIs) are the go-to pharmacological options. However, it’s worth noting that psychotherapy often requires a long-term commitment, whereas the anxiolytic effects of antidepressant medications typically emerge around 2–4 weeks after starting treatment (Jakubovski et al., 2019). This delay can result in patients experiencing adverse effects before experiencing the therapeutic benefits (Szuhany and Simon, 2022). In this context, the exploration of novel approaches to treating people with anxiety is an area that has received limited attention (Alonso et al., 2018).

Electroacupuncture (EA) is a modernized adaptation of traditional Chinese medicine’s acupuncture, a technique known for its significant impact on the nervous, endocrine, and immune systems (Li et al., 2013). EA shares a similar mechanism with acupuncture but is more accessible and user-friendly (Liu et al., 2017; Chen et al., 2018). Extensive research has already highlighted the positive effects of EA in alleviating symptoms associated with various neuropsychiatric conditions (Li X. et al., 2019; Tamtaji et al., 2019; Wang et al., 2019). Furthermore, animal studies have demonstrated the capacity of EA to ameliorate behaviors related to anxiety, and clinical studies have confirmed its anxiolytic effects (Amorim et al., 2022; Wu et al., 2022; Xu et al., 2023). Collectively, these findings suggest that EA treatment could serve as a promising complementary therapy for anxiety disorders. However, the precise mechanisms behind its effectiveness require further exploration.

The gut-brain axis, representing the bidirectional connection between the gut microbiota and the central nervous system (CNS), is a crucial player in regulating emotions and stress. It operates through various mechanisms, including metabolic, neural, hormonal, and immune-mediated pathways (Foster et al., 2017; Rieder et al., 2017). A substantial body of evidence has linked dysbacteriosis (imbalances in gut microbiota) to psychiatric symptoms like anxiety and depression (Foster and Neufeld, 2013; Simpson et al., 2021). For instance, the configuration of gut microbiota undergoes significant changes in individuals with anxiety disorders, and alterations in specific microbiota are associated with anxiety symptoms (Chen et al., 2019; Nikolova et al., 2021). Moreover, preclinical research has highlighted disorders in the gut microbiota of rodents showing anxiety-like behaviors, and these disruptions were corrected after administering bacterial probiotics (Mayer et al., 2015). Another study demonstrated that transplanting fecal microbiota from individuals with depression into mice treated with antibiotics led to anxiety-like behavior (Zhang et al., 2022). Consequently, the gut microbiota’s role appears to be closely linked to the development of anxiety. Significantly, prior studies have reported that EA can mitigate myocardial ischemia–reperfusion lesions in rats by modulating gut microbiota and restore imbalances in cecal microbiota in mice that were obese as a consequence of a diet high in fat (Bai et al., 2021; Xia et al., 2022). This suggests that the regulation of gut microbiota could be one of the potential mechanisms underlying EA’s effects. However, most research on anxiety models has primarily focused on the genus level in microbiota analysis, which leaves the impact of EA on the gut microbiota at species level of mouse models of anxiety still unclear.

In this context, our current study aimed to investigate how EA treatment impacts the gut microbiome in a murine model of CRS. This is a well-established and widely accepted stress-related anxiety model that reliably induces anxiety-like behavior (Liu et al., 2020; Yan et al., 2022). Simultaneously, we examined the impact of CRS on the gut microbiota at a species-specific level.

## 2. Materials and methods

### 2.1. Animals

Male C57BL/6 mice, aged 8 weeks and with a weight between 18 and 22 g, were supplied by the Animal Center of Air Force Military Medical University. These mice were group-housed in cages, with each cage accommodating four mice. They had unrestricted access to food and water and were kept in a controlled environment at a temperature of 20–25°C. The cages had wire bottoms, and the mice followed a 12-h light/dark cycle, with lights on from 8:00 a.m. to 8:00 p.m. The research procedures conducted in this study received approval from the Ethics Committee of Xi’an Gaoxin Hospital under the reference number 2023-GXKY-0011. All experiments were conducted in accordance with the guidelines provided in the National Institutes of Health Guide for the Care and Use of Laboratory Animals.

### 2.2. Experimental design

To investigate the impact of CRS on the gut microbiome effectively, mice underwent a 7-day acclimatization period before being randomly assigned to two groups: Control (*n* = 8) and CRS (*n* = 16). In the CRS group, mice were exposed to CRS for 2 h per day over a continuous 14-day period. This involved placing the mice in conical tubes (50 mL) equipped with airflow holes. In contrast, the Control group mice were transferred from their original cage to an experimental room, delicately managed for 5 min, and then transported back to their holding place 2 h later. This procedure was repeated over the course of 14 days (Liu et al., 2020; Yan et al., 2022). Behavioral tests were administered 24 h after CRS procedure, and stool samples were gathered and preserved in liquid nitrogen before the behavioral assessments. To assess how EA could impact the gut microbiome within the CRS model, another group of mice, after a 7-day adaptation period, were randomly assigned to three groups: Sham, CRS + fEA, and CRS + EA, each consisting of 8 mice. In the Sham group, mice received same treatment to the Control group for 14 days. Subsequently, they received false EA stimulation during 7 days. In the CRS + fEA group and CRS + EA groups, mice underwent 14 days of CRS, after which they received either false EA stimulation or real EA stimulation for 7 days, respectively. Behavioral tests were administered 24 h after the last EA procedure, and stool samples were gathered and preserved in liquid nitrogen before the behavioral assessments.

### 2.3. EA treatment

The EA treatment involved a daily session lasting 30 min, with a frequency of 2/15 Hz, intensity set at 1 mA, and a dilatational waveform. We employed the G6805–2 EA instrument (Serial Number: 227033; Qingdao Xinsheng Ltd.) for the EA procedures. Consistent with previous protocols (Zhou et al., 2022), mice received anesthesia in the form of isoflurane at a concentration of 1.5 MAC while undergoing the EA application. To complete the electrical circuit, one end of the acupuncture needle was inserted into the acupoint “Bai hui” (GV20), while the other end was connected to an electrode clamped onto the tail. For the sham stimulation, the same acupoint was used, but without the application of electricity.

### 2.4. Open-field test (OFT)

The open field chamber used in this study was constructed from white polycarbonate and measured 50 cm × 50 cm in size. Each mouse was positioned in the middle of the chamber, and their behavior was monitored and recorded for a duration of 5 min. We utilized an overhead video-tracking and analysis system acquired from Top Scan, Clever Sys Inc. (United States) for this purpose. Specifically, we measured the time that mice spent within the central area, which was a 25 cm × 25 cm square at the center of the chamber, as well as the whole distance they covered during the observation period.

### 2.5. Elevated-plus maze test (EPMT)

The maze apparatus used in our study, supplied by Dig Behav, Ji Liang Co. Ltd. (China) was comprised of two open arms (each measuring 35 cm × 6 cm) and two enclosed arms (each measuring 35 cm × 6 cm), elevated 50 cm over the floor. During the testing phase, mice were initially positioned in the central square of the maze, oriented toward one of the open arms. Their behavior was then observed and recorded for 5 min. We quantified the number of entries into the open arms (entry count) and the duration of placement within the open arms, employing the same monitoring system as used in the OFT. All tests occurred in low light conditions, and the test area was thoroughly sanitized with 30% ethanol after each trial.

### 2.6. Gathering of fecal samples and sequencing of 16S rRNA microbiome

Each mouse was placed in a metabolic cage between 7:00 a.m. and 11:00 a.m., and fecal samples were collected in sterile cryotubes, and immediately frozen in liquid nitrogen before further analysis. Undefecated mice promote defecation by lifting their tails, ensuring that each mouse collects at least one fecal sample. Genomic DNA was then obtained from these fecal samples using the E.Z.N.A. Stool DNA Kit, manufactured by Omega Bio-Tek, United States (Yan et al., 2020). For the amplification of bacterial 16S rRNA genes, we employed universal bacterial primers 1492R (5’-RGYTACCTTGTTACGACTT-3′) and 27F (5’-AGRGTTYGATYMTGGCTCAG-3′) (Yu et al., 2013). The purified DNA products were combined in equal amounts, and a DNA library was created following the guidelines provided by PacBio, utilizing the SMRTbell prep kit 3.0 (Pacific Biosciences, CA, United States). Then, these purified SMRTbell libraries underwent sequencing on the PacBio Sequel IIe System, also from Pacific Biosciences, CA, United States, with the sequencing being conducted by Majorbio Bio-Pharm Technology Co. Ltd. based in Shanghai, China. Analysis of the raw FASTQ files was carried out using USEARCH 8.0. The sequences were clustered into operational taxonomic units (OTUs) utilizing UPARSE, version 7.1,^1^ with a 97% pairwise identity threshold. Taxonomic classifications were determined using the ribosomal database project classifier algorithm, available at http://rdp.cme.msu.edu/. Following the OTU assignment and taxonomy analysis, subsequent analyses including Alpha Diversity Analysis, Linear Discriminant Analysis (LDA), Principal Coordinate Analysis (PCoA), and correlation analysis were performed using the Majorbio cloud platform, which is provided by Majorbio Bio-Pharm Technology Co., Ltd.

### 2.7. Statistical analysis

Statistical analyses were performed with the GraphPad v.8.0 or SPSS 21.0 software (IBM-SPSS Inc., United States). We first assessed the normal distribution of continuous data using the Shapiro–Wilk test. If the data met the criteria for normal distribution or variance homogeneity, we conducted unpaired t-tests or one-way analysis of variance (ANOVA), and subsequent Bonferroni post-hoc tests for pairwise comparisons. Alternatively, if the data did not meet these criteria, nonparametric tests such as the Wilcoxon rank-sum test or Kruskal-Wallis test were applied. To examine correlations between behaviors and gut microbiota at the species level, we utilized Spearman’s rank correlation coefficient. All significance tests were two-tailed, and a significance level of *p* < 0.05 was deemed statistically significant.

## 3. Results

### 3.1. CRS induces behaviors related to anxiety and alterations in the gut microbiota diversity

**Figure 1:**
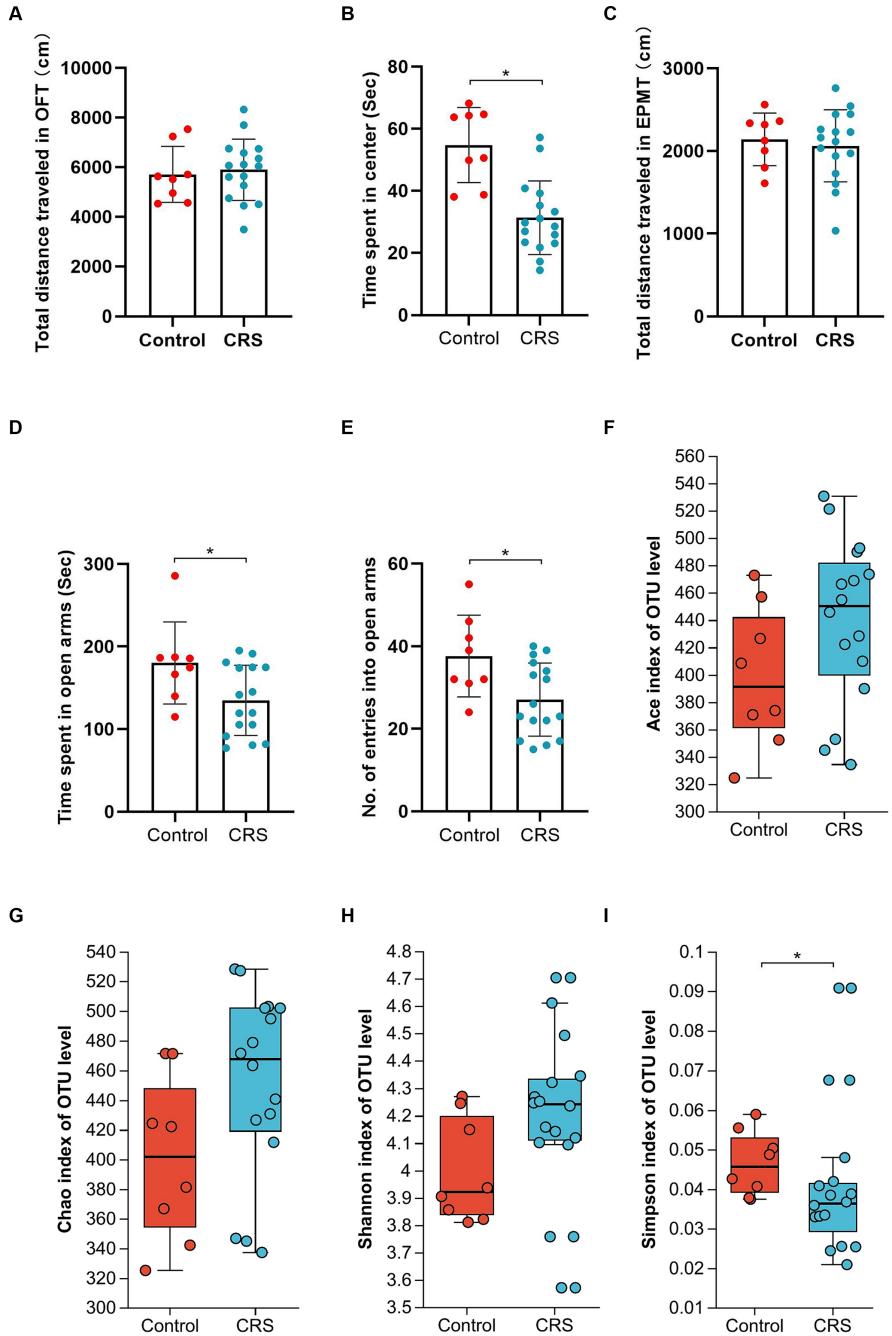
CRS induces anxiety-like behaviors and partly influences the α-diversity of gut microbiota. **(A)** The total distance traveled in the OFT. **(B)** The time spent in center area of OFT. **(C)** The total distance traveled in the EPMT. **(D)** Time spent in open arms of EPMT. **(E)** The no. of entries into the open arms of EPMT. Bar chart displays means and standard error for each group. Box plots depicted the indices of **(F)** Ace, **(G)** Chao, **(H)** Shannon and **(I)** Simpson of OTU level. The horizontal lines in the box plots represent median values; upper and lower ranges of the box represent the 75% and 25% quartiles. The red and blue circle represents one value from individual mice in Control group or CRS group, respectively. *p < 0.05.

**Figure 2:**
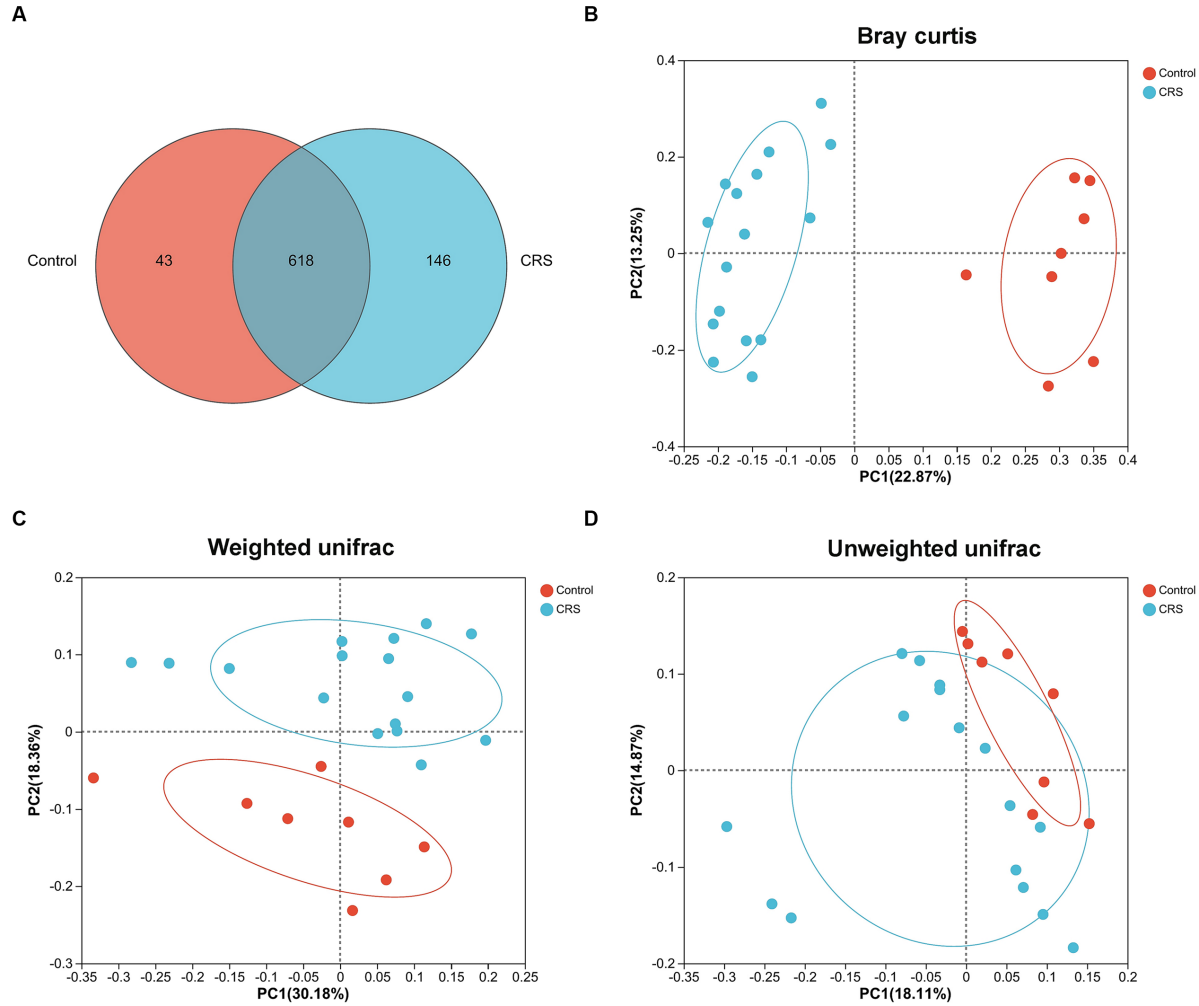
CRS influences the β-diversity of gut microbiota. **(A)** The number of common and unique OTUs between Control and CRS is displayed by the Venn diagram. PCoA plots of bacterial beta-diversity based on **(B)** Bray curtis, **(C)** Weighted UniFrac and **(D)** Unweighted UniFrac distance. The red or blue circle represents one value from individual mice in Control group or CRS group, respectively.

No significant difference was observed in the whole distance traveled in the OFT (*t* = 0.355, df = 22, *p* = 0.726; Figure 1A) and total distance traveled in the EPMT (*t* = 0.443, df = 22, *p* = 0.662; Figure 1C) between the groups. However, the CRS group exhibited reduced time spent in the center area of the OFT (*t* = 4.535, df = 22, *p* < 0.001; Figure 1B), decreased time spent in the open arms of the EPMT (*t* = 2.321, df = 22, *p* = 0.03; Figure 1D) and fewer entries into the open arms of the EPMT (*t* = 2.654, df = 22, *p* = 0.015; Figure 1E) compared to the Control group. Moreover, we compared α-diversity results by Wilcoxon rank-sum test and found there were no significant differences in the Ace, Chao and Shannon index (Figures 1F–H), except for the Simpson index (*p* = 0.04, Figure 1I). Subsequent analysis revealed 661 OTUs at the level of species in the Control group and 764 OTUs in the CRS group (Figure 2A). Additionally, β-diversity analysis showed that fecal microbiomes were separated into two groups according to the composition of microbial communities by Bray-Curtis (*r*^2^ = 0.258, *p* = 0.001; Figure 2B), weighted UniFrac (*r*^2^ = 0.281, *p* = 0.001; Figure 2C) and unweighted UniFrac (*r*^2^ = 0.124, *p* = 0.001; Figure 2D) analyses. Together, CRS induces anxiety-like behaviors and leads to significant changes in the β-diversity but not α-diversity.

### 3.2. CRS alters the composition of gut microbiota

**Figure 3:**
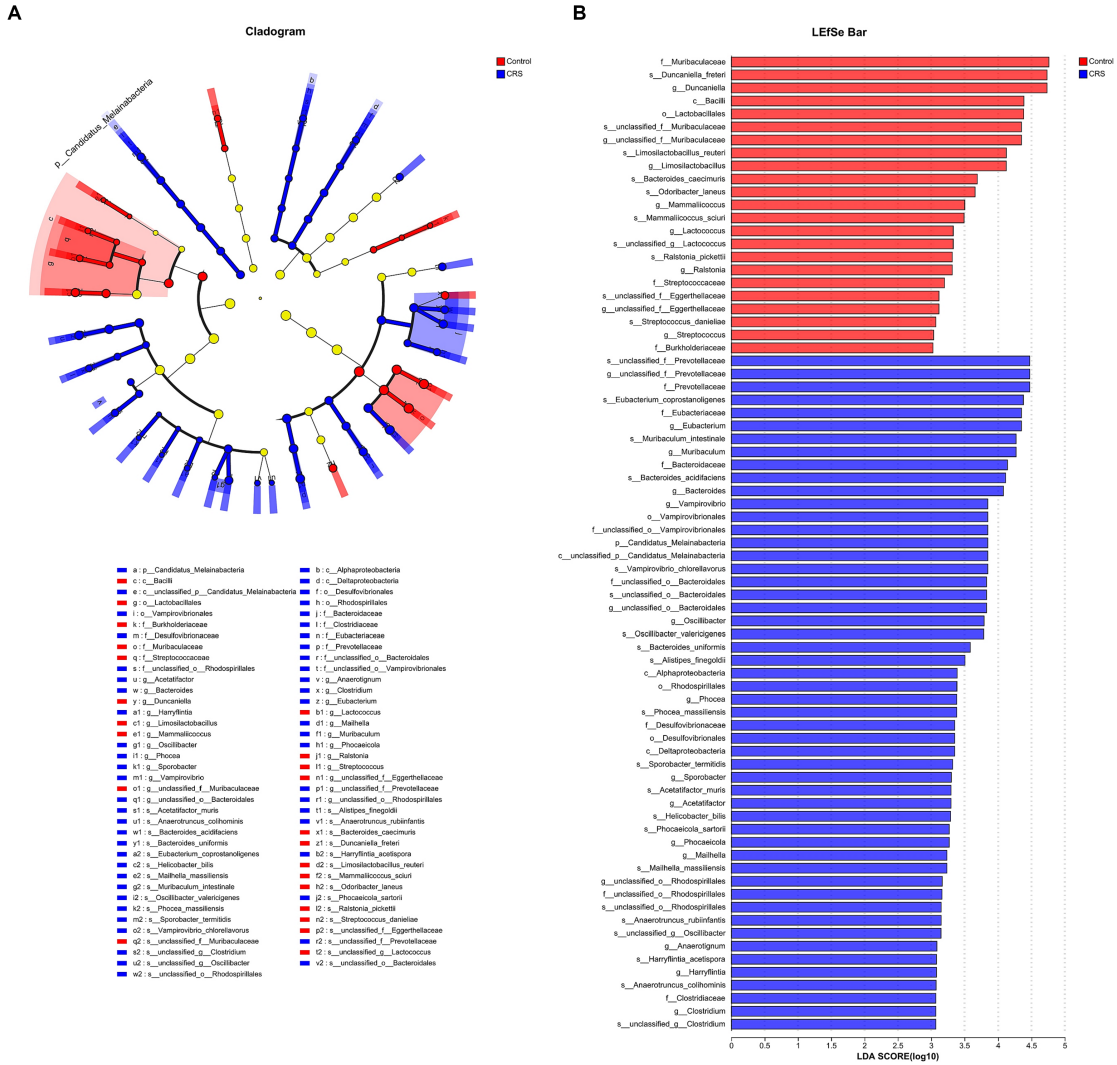
CRS influences taxonomic composition of gut microbiota. **(A)** The taxonomic cladogram shows the bacterial taxa enriched in Control (red dots) and CRS (blue dots). Yellow dots represent microbial taxa that did not significantly affect the differences between each group. **(B)** The LDA discriminant bar chart shows the microbial taxa with significant differences in the Control (red) and CRS (blue) groups. Larger LDA scores represent a greater effect of species abundance on the different effects. Only taxa with an LDA score > 3.0 are shown in the figure.

We found distinctions in taxonomic composition between the Control and CRS groups using both LDA with a threshold of LDA ≥ 3 and a significance level of *p* < 0.05, and Effect Size (LEfSe) analysis, as illustrated in Figure 3. The relative abundance of the families *Muribaculaceae*, *Streptococcaceae* and *Burkholderiaceae*; genus *Duncaniella*, *unclassified_f_Muribaculaceae*, *Limosilactobacillus*, *Mammaliicoccus*, *Lactococcus*, *Ralstonia*, *unclassified_f_Eggerthellaceae* and *Streptococcus*; species *Ralstonia_pickettii*, *Streptococcus_danieliae*, *unclassified_g_Lactococcus*, *Limosilactobacillus_reuteri*, *Mammaliicoccus_sciuri*, *Odoribacter_laneus*, *unclassified_f_Muribaculaceae*, *Duncaniella_freteri*, *Bacteroides_caecimuris* and *unclassified_f_Eggerthellaceae* were enriched in the Control group. Whereas phylum *Candidatus_Melainabacteria*; family *Bacteroidaceae*, *Prevotellaceae*, *unclassified_o_Bacteroidales*, *unclassified_o_Vampirovibrionales*, *Clostridiaceae*, *Eubacteriaceae*, *unclassified_o_Rhodospirillales* and *Desulfovibrionaceae*; genus *unclassified_f_Prevotellaceae*, *Eubacterium*, *Muribaculum*, *Bacteroides*, *Vampirovibrio*, *unclassified_o_Bacteroidales*, *Oscillibacter*, *Phocea*, *Sporobacter*, *Acetatifactor*, *Phocaeicola*, *Mailhella*, *unclassified_o_Rhodospirillales*, *Anaerotignum*, *Harryflintia* and *Clostridium*; species *Harryflintia_acetispora*, *Bacteroides_acidifaciens*, *Bacteroides_uniformis*, *Phocaeicola_sartorii*, *Muribaculum_intestinale*, *unclassified_f_Prevotellaceae*, *Alistipes_finegoldii*, *unclassified_o_Bacteroidales*, *Vampirovibrio_chlorellavorus*, *unclassified_g_Clostridium*, *Eubacterium_coprostanoligenes*, *Acetatifactor_muris*, *Anaerotruncus_colihominis*, *Anaerotruncus_rubiinfantis*, *Oscillibacter_valericigenes*, *unclassified_g_Oscillibacter*, *Phocea_massiliensis*, *Sporobacter_termitidis*, *unclassified_o_Rhodospirillales*, *Mailhella_massiliensis* and *Helicobacter_bilis* were enriched in the CRS group.

**Figure 4:**
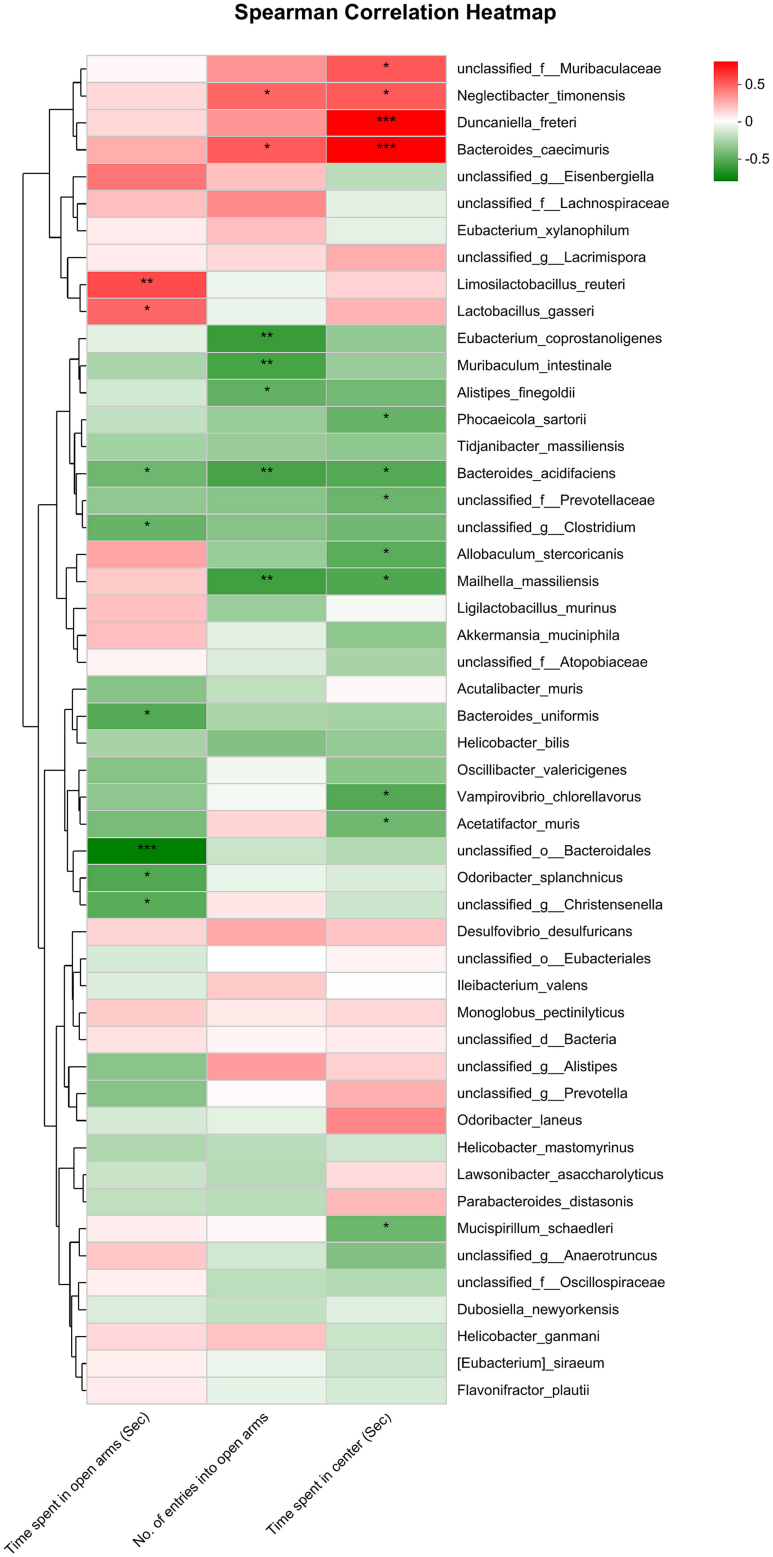
Correlation between anxiety-like behaviors (represented by the time spent in open arms of EPMT, no. of entries into the open arms of EPMT and time spent in center area of OFT) and the differential gut microbiota species between Control and CRS. Red and green squares indicate positive and negative correlations, respectively, and the intensities of the colors are proportional to the degree of correlation. *p < 0.05; **p < 0.01; **p < 0.001.

The analysis exploring the relationship between anxiety-related behaviors and gut microbiota at the species level revealed a significant negative correlation. Specifically, the time spent in open arms exhibited an inverse relationship with the abundance of *unclassified_o_Bacteroidales*, *unclassified_g_Christensenella*, *Odoribacter_splanchnicus*, *Acutalibacter_muris*, *Bacteroides_uniformis*, *Oscillibacter_valericigenes* and *Acetatifactor_muris*; but had a positive correlation with the abundance of *Lactobacillus_gasseri*, *Limosilactobacillus_reuteri*, *Akkermansia_muciniphila* and *Ligilactobacillus_murinus*. The No. of entries into open arms had a negative correlation with the abundance of *Bacteroides_acidifaciens* and *Eubacterium_coprostanoligenes*. The time spent in center had a negative correlation with the abundance of *unclassified_g_Anaerotruncus*, *Phocaeicola_sartorii*, *unclassified_f_Prevotellaceae*, *Mailhella_massiliensis*, *Vampirovibrio_chlorellavorus*, *Bacteroides_acidifaciens*, *Bacteroides_uniformis* and *Acetatifactor_muris;* whereas it had a positive correlation with the abundance of *unclassified_f_Muribaculaceae*, *Duncaniella_freteri*, *Bacteroides_caecimuris* and *Lactobacillus_gasseri* (Figure 4).

### 3.3. EA ameliorates behaviors related to anxiety in mice under CRS

**Figure 5:**
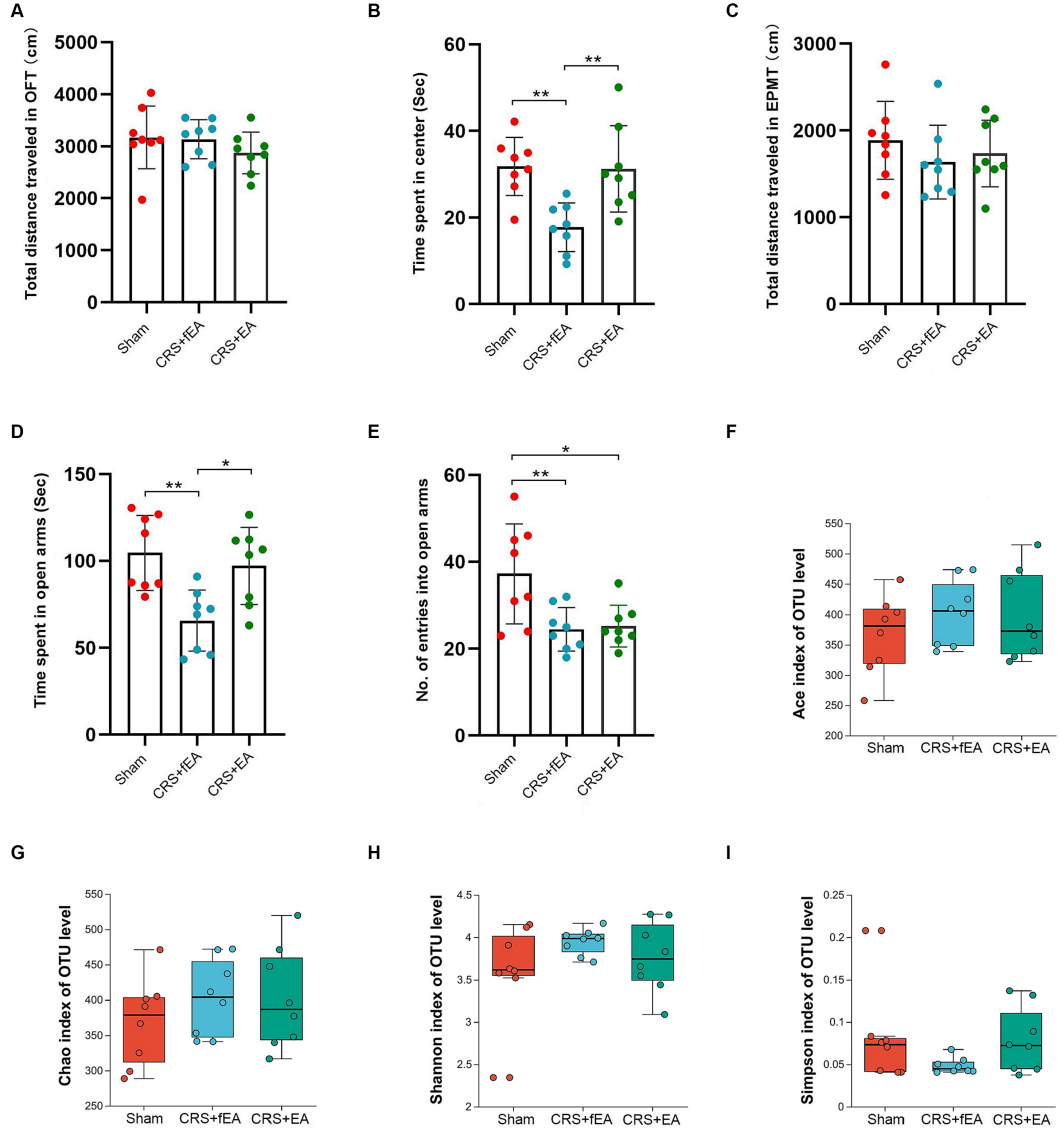
EA partly ameliorates CRS-induced anxiety-like behaviors but did not influence the α-diversity of gut microbiota. **(A)** The total distance traveled in the OFT. **(B)** The time spent in center area of OFT. **(C)** The total distance traveled in the EPMT. **(D)** Time spent in open arms of EPMT. **(E)** The no. of entries into the open arms of EPMT. Bar chart displays means and standard error for each group. Box plots depicted the indices of **(F)** Ace, **(G)** Chao, **(H)** Shannon and **(I)** Simpson of OTU level. The horizontal lines in the box plots represent median values; upper and lower ranges of the box represent the 75% and 25% quartiles. The red, blue and green circle represents one value from individual mice in Sham, CRS + fEA and CRS + EA group, respectively. *p < 0.05; **p < 0.01.

As depicted in Figure 5, no differences were observed among the Sham, CRS + fEA, and CRS + EA group in terms of the whole distance traveled in the OFT (*F_2, 21_* = 0.954, *p* = 0.401; Figure 5A) and total distance traveled in the EPMT (*F_2, 21_* = 0.726, *p* = 0.495; Figure 5C). However, notable differences were evident among the three groups in the time they remained in the center area of the OFT (*F_2, 21_* = 8.653, *p* < 0.01; Figure 5B), the time they remained in the open arms of the EPMT (*F_2, 21_* = 8.086, *p* < 0.01; Figure 5D), and the number of entries into the open arms of the EPMT (*F_2, 21_* = 6.820, *p* = 0.005; Figure 5E). *Post hoc* comparisons provided additional insights into the effects of CRS on behavior. Specifically, CRS resulted in a reduction in the time the mice stayed in the center area of the OFT, as well as the time they remained in the open arms and the number of entries into the open arms in the EPMT when compared to the Sham group (*p* < 0.01). Notably, the application of EA (CRS + EA) partially mitigated the anxiety-like behavior induced by CRS in mice, as indicated by an increase in the time the animals stayed in the center area of the OFT and the open arms of the EPMT when compared to the CRS + fEA group (*p* < 0.05).

### 3.4. EA influences the composition of gut microbiota in CRS-treated mice

**Figure 6:**
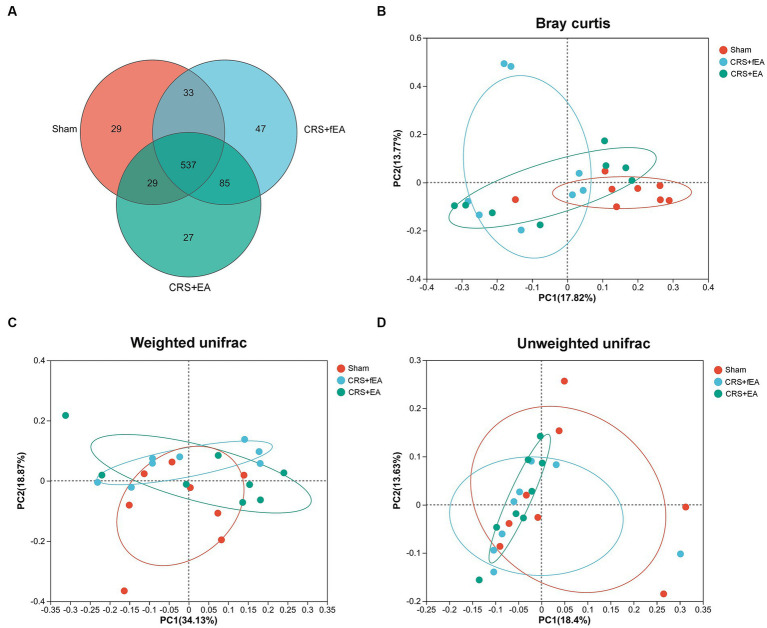
EA partly influences the β-diversity of gut microbiota in CRS-treated mice. **(A)** The number of common and unique OTUs among the three groups is displayed by the Venn diagram. **(B–D)** PCoA plots of bacterial beta-diversity based on **(B)** Bray curtis, **(C)** Weighted UniFrac and **(D)** Unweighted UniFrac distance. The red, blue and green circle represents one value from individual mice in Sham, CRS + fEA and CRS + EA group, respectively.

**Figure 7:**
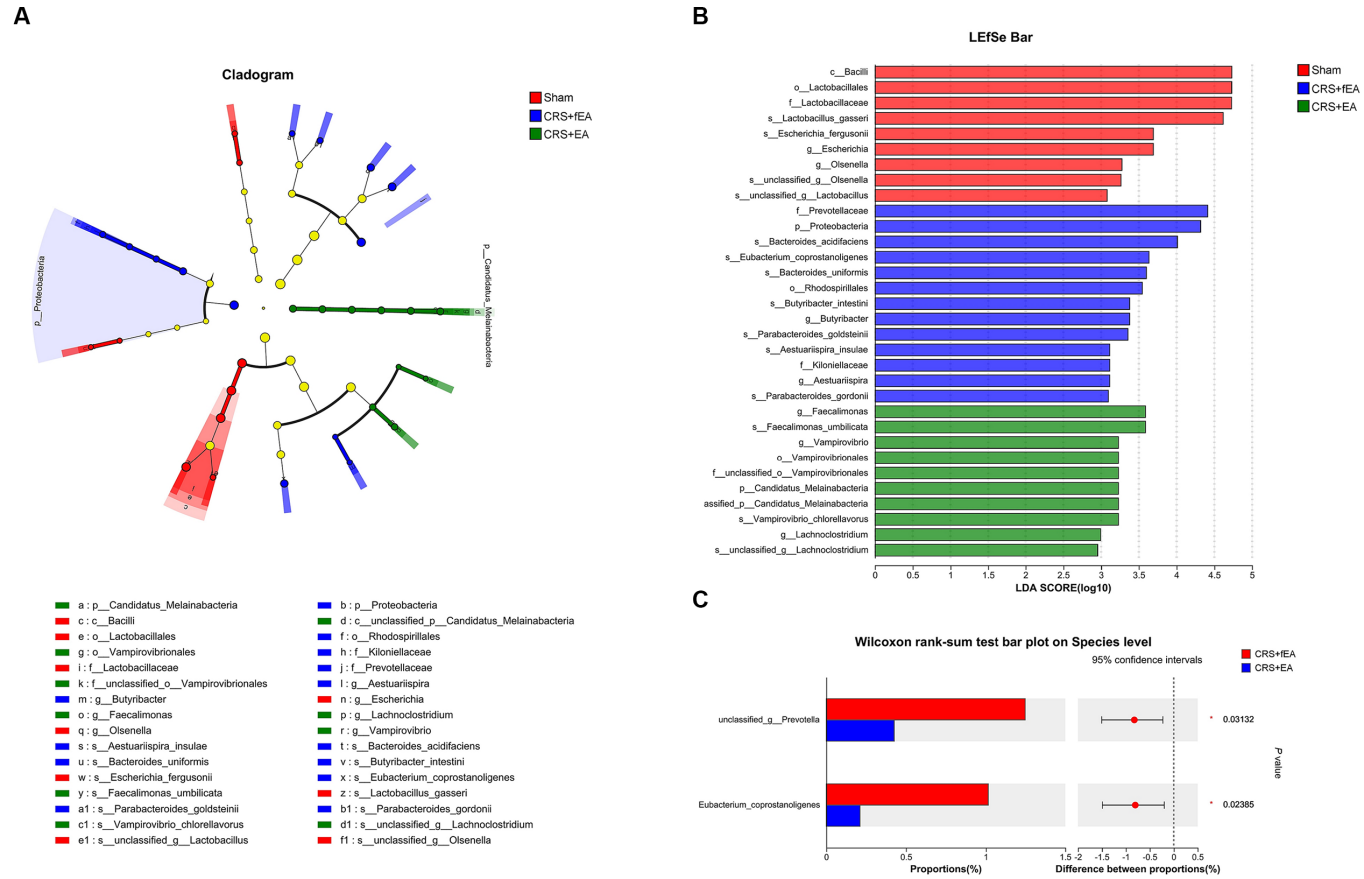
EA influences taxonomic composition of gut microbiota in CRS-treated mice. **(A)** The taxonomic cladogram shows the bacterial taxa enriched in Sham (red dots), CRS + fEA (blue dots) and CRS + EA (Green dots). Yellow dots represent microbial taxa that did not significantly affect the differences between each group. **(B)** The LDA discriminant bar chart shows the microbial taxa with significant differences in each group. Only taxa with an LDA score > 2.0 are shown in the figure. **(C)** Wilcoxon rank-sum test showed the changed species between CRS + fEA (red) and CRS + EA (blue) groups.

Kruskal-Wallis test found that there were no significant differences in the α-diversity indexes among Sham, CRS and CRS + EA group, including Ace (Figure 5F), Chao (Figure 5G), Shannon (Figure 5H), and Simpson (Figure 5I). Following downstream analysis, we found that the Sham group yielded 628 OTUs at the level of species, the CRS + fEA group produced 702 OTUs, and the CRS + EA group resulted in 678 OTUs (Figure 6A). Furthermore, in the β-diversity analysis, we observed that the fecal microbiomes exhibited division into three distinct clusters solely based on microbial community composition, as determined by the Bray-Curtis metric (*r*^2^ = 0.131, *p* = 0.012; Figure 6B). However, we did not find significant differences in the weighted UniFrac (*r*^2^ = 0.112, *p* = 0.159; Figure 6C) and unweighted UniFrac (*r*^2^ = 0.108, *p* = 0.117; Figure 6D) analyses. Consequently, it appears that EA treatment had a partial influence on β-diversity while not affecting α-diversity. Furthermore, in the Sham group, we observed an enrichment in the relative abundance of the the family *Lactobacillaceae*, genus *Escherichia* and *Olsenella*, species *unclassified_g_Lactobacillus*, *unclassified_g_Olsenella*, *Escherichia_fergusonii* and *Lactobacillus_gasseri*. Meanwhile, phylum *Proteobacteria*; family *Kiloniellaceae*; genus *Butyribacter* and *Aestuariispira*; species *Parabacteroides_gordonii*, *Aestuariispira_insulae*, *Parabacteroides_goldsteinii*, *Butyribacter_intestini*, *Bacteroides_uniformis*, *Eubacterium_coprostanoligenes* and *Bacteroides_acidifaciens* were enriched in the CRS + fEA group. Phylum *Candidatus_Melainabacteria*; family *Prevotellaceae* and *unclassified_o_Vampirovibrionales*; genus *Faecalimonas*, *Vampirovibrio* and *Lachnoclostridium*; species *Faecalimonas_umbilicata*, *Vampirovibrio_chlorellavorus* and *unclassified_g_Lachnoclostridium* were enriched in CRS + EA group (LDA ≥ 2, *p* < 0.05; Figures 7A,B). Notably, only *unclassified_g_Prevotella* and *Eubacterium_coprostanoligenes* were enriched in the CRS + fEA group compared to the CRS + EA group (Figure 7C).

**Figure 8:**
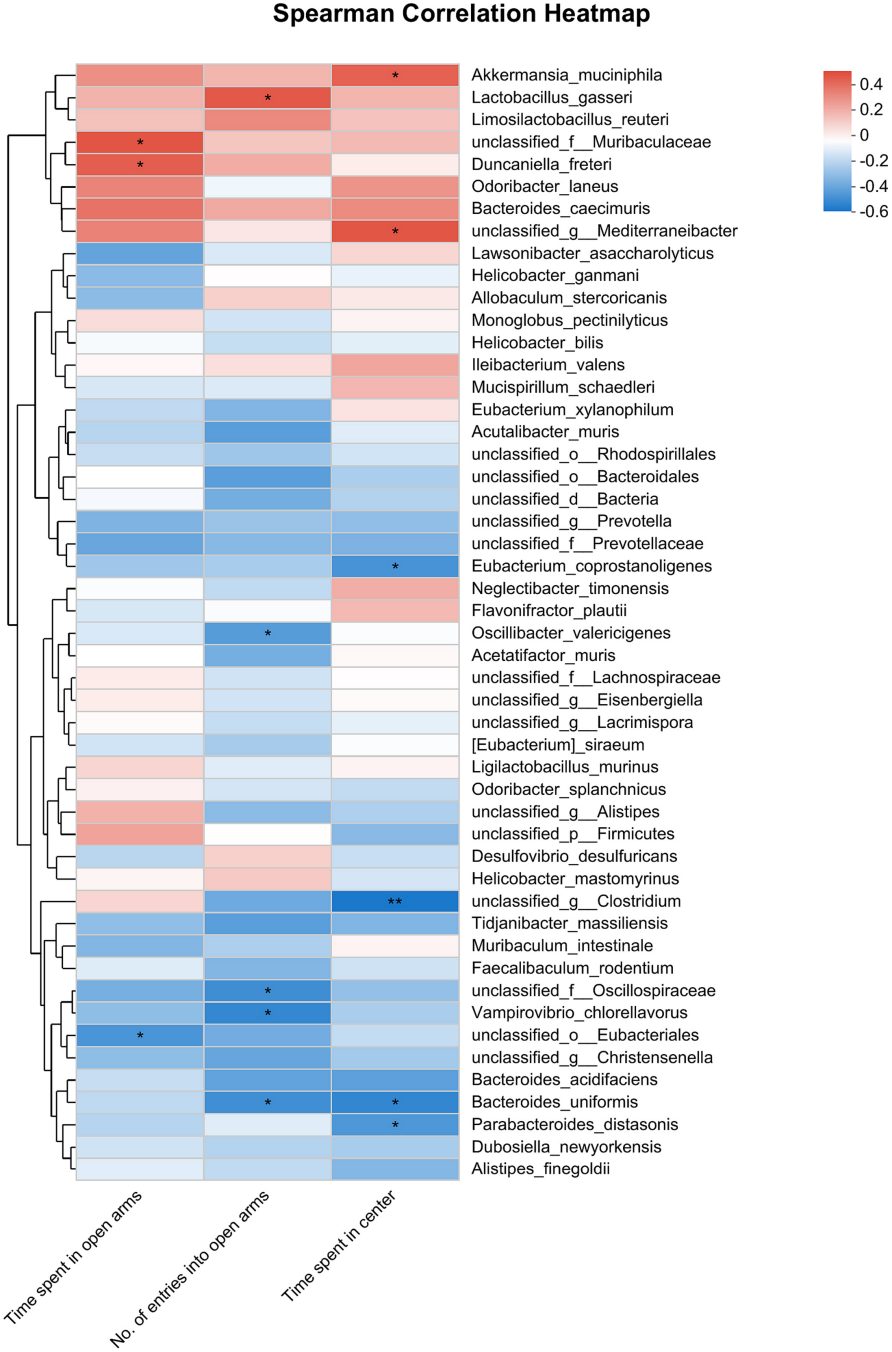
Correlation between anxiety-like behaviors (represented by the time spent in open arms of EPMT, no. of entries into the open arms of EPMT and time spent in center area of OFT) and the differential gut microbiota species among Sham, CRS + fEA and CRS + EA group. Red and blue squares indicate positive and negative correlations, respectively, and the intensities of the colors are proportional to the degree of correlation. *p < 0.05; **p < 0.01.

The correlation analysis that evaluated anxiety-like behaviors and gut microbiota at the species level provided further insights. Specifically, the time spent in open arms exhibited a negative correlation with the abundance of *unclassified_o_Eubacteriales* but showed a positive correlation with the abundance of *unclassified_f_Muribaculaceae* and *Duncaniella_freteri*. Furthermore, the number of entries into open arms displayed a negative correlation with the abundance of *Bacteroides_uniformis*, *unclassified_f_Oscillospiraceae*, *Vampirovibrio_chlorellavorus*, and *Oscillibacter_valericigenes*, while it showed a positive correlation with the abundance of *Lactobacillus_gasseri*. Lastly, the time the mice stayed in the center area had a negative correlation with the abundance of *Parabacteroides_distasonis*, *Bacteroides_uniformis*, and *Eubacterium_coprostanoligenes*, but it exhibited a positive correlation with the abundance of *unclassified_g_Mediterraneibacter* and *Akkermansia_muciniphila* (Figure 8).

## 4. Discussion

The current study provides insights into the alleviation of anxiety-like behaviors in mice with CRS through EA treatment. Additionally, we employed full-length 16S rRNA microbiome sequencing to delineate, for the first time, the unique gut microbial signatures at the species level associated with CRS and EA treatment. Furthermore, we investigated the connection that could link specific gut microbial species and behaviors related to anxiety. Beyond shedding light on the gut microbiota imbalances related to anxiety, these findings may contribute to a deeper understanding of the potential mechanisms underlying the anti-anxiety effects of EA.

Anxiety disorders represent the most prevalent category of mental illnesses, encompassing conditions such as generalized anxiety disorder (GAD), separation anxiety, selective mutism, specific phobias, social anxiety disorder (SAD), panic disorder, and agoraphobia (Penninx et al., 2021). The link between dysbacteriosis (microbial imbalances) and anxiety disorders, spanning the spectrum of anxiety conditions (Stanislawski et al., 2021) including specific subtypes like GAD (Chen et al., 2019) and SAD (Butler et al., 2023), has been extensively studied. Additionally, preclinical research has delved into the influence of gut microbiota on behaviors related to anxiety (Neufeld et al., 2011). For instance, the absence of gut microbiota has been shown to heighten anxiety-like responses to acute stress in rats, and fecal transplantation has been observed to impact anxiety-like behaviors in recipient mice (Crumeyrolle-Arias et al., 2014; Li N. et al., 2019). Nevertheless, the effects of CRS, a relatively reliable method for inducing anxiety-like behavior through 2-h daily exposure for 14 consecutive days (Yan et al., 2022), on the gut microbiome, especially at the species level, remain to be fully elucidated. This study reveals that 8 genera showed enrichment in the Control group, while 16 genera proved enrichment in the CRS group. Additionally, we identified 10 species enriched in the Control group and 21 species enriched in the CRS group. This result is similar to the study by Yang et al., who also found that the relative abundances of *Lachnospiraceae*, *Lachnospirales*, *Lachnospiraceae_NK4A136_group* and *Clostridia* were higher in CRS mice than in naive mice (Yang Q. X. et al., 2022). Given that the functions of these species still need further exploration, it is difficult to say whether these bacteria are beneficial or harmful. Among these species, *unclassified_o_Bacteroidales*, *Limosilactobacillus_reuteri*, *unclassified_f_Muribaculaceae*, *Duncaniella_freteri*, and *Bacteroides_caecimuris* were more abundant in the Control group and showed a negative correlation with anxiety-like behaviors. Conversely, *Bacteroides_uniformis*, *Oscillibacter_valericigenes*, *Acetatifactor_muris*, *Bacteroides_acidifaciens*, *Phocaeicola_sartorii*, *unclassified_f_Prevotellaceae*, *Mailhella_massiliensis*, and *Vampirovibrio_chlorellavorus* were more abundant in the CRS group and exhibited a positive correlation with anxiety-like behaviors. These findings suggest that CRS induces substantial dysbiosis, and alterations in these 13 species are associated with CRS-induced anxiety-like behaviors. Nonetheless, the impact and underlying mechanisms of these microbial populations on anxiety-like behavior warrant further validation through bacterial transplantation studies.

EA is an alternative therapeutic approach that combines traditional acupuncture techniques with electrotherapy and is increasingly recognized for its potential in treating neuropsychiatric disorders. A growing body of research has highlighted the beneficial effects of EA, particularly when applied at the “Bai hui” (GV20) acupoint. Studies have shown that GV20-based EA can regulate basic fibroblast growth factor (FGF2) in the rat hippocampus (Yao et al., 2021), enhance the phosphatidylinositol-3-kinase (PI3K)/protein kinase B (Akt) pathway in a rat model of cerebral ischemia/reperfusion injury (Wang et al., 2021), and reduce microglial triggering while decreasing levels of IL-1β and IL-6 in cerebral tissue in aged mice (Sun et al., 2022). Importantly, these mechanisms have been implicated in the development of anxiety (Di Liberto et al., 2017; Liu et al., 2022). Furthermore, previous research has indicated that EA treatment, specifically when applied at the “Bai hui” acupoint using a dilatational wave (2/15 Hz, 1 mA), can effectively mitigate behaviors related to anxiety in rodent models of post-traumatic stress disorder (Zhou et al., 2019, 2022). In line with these findings, our study demonstrates that EA treatment with these parameters at the “Bai hui” acupoint increases the time the subjects stayed in the center area of the OFT and open arms of the EPMT, which are two key indicators of anxiety-like behaviors (Komada et al., 2008), indicating that EA treatment applied at “Bai hui” might be an effective intervention for CRS-induced anxiety-like behavior.

Bidirectional communication in the microbiota-gut-brain axis (MGBA) has been well-declared (Mayer, 2011; Dinan and Cryan, 2012; Foster and Neufeld, 2013), This intricate network involves multiple components, including the hypothalamic–pituitary–adrenal (HPA) axis, the vagal nerve, endocrine signaling, immune mediators, and the generation of bacterial metabolites (Goralczyk-Binkowska et al., 2022). Consequently, gut microbiota can influence cerebral function by impacting inflammation signaling and producing metabolites (Agirman et al., 2021). At the same time, the brain has the capacity to regulate the gastrointestinal tract and the enteric nervous system, thereby exerting an influence on the composition of the gut microbiota (Rhee et al., 2009). In this context, neuroregulatory techniques like repetitive transcranial magnetic stimulation (rTMS) (Zhou et al., 2023), vagus nerve stimulation (VNS) (Campbell et al., 2016) and transcranial direct current stimulation (tDCS) (Artifon et al., 2020) have been reported to affect the gut microbiota. Similarly, EA could also influence the composition and activity of gut microbiota. Recent randomized controlled trials have revealed that EA can enlarge the relative abundances of *Parasutterella* and *Bacteroides* while decreasing the relative abundances of *Dialister*, *Hungatella*, *Megasphaera*, *Barnesiella*, *Allisonella*, *Intestinimon* and *Moryella* at the genus level in the treatment of Parkinson’s disease (Nazarova et al., 2022). Preclinical studies have demonstrated that EA attenuates delirium-like behavior induced by surgical pain in mice by remodeling gut microbiota and regulates gut microbiota in ischemic stroke mice through the brain-gut axis (Yang L. Y. et al., 2022; Zhang et al., 2023). However, whether EA treatment can influence gut microbiota in animal models of anxiety remains largely unexplored.

Our present study delved into the composition of the gut microbiome in mice subjected to CRS modeling after EA treatment. Similar to other neuromodulation therapies (Korenblik et al., 2023), EA had a partial effect on β-diversity (measured by Bray-Curtis) but did not influence α-diversity. Generally, the abundance of α-diversity was positively correlated with health status. Yang et al. reported that α-diversity, including the Chao, Shannon and observed species index, was significantly decreased after CRS (Yang et al., 2022). However, other works found that there was no significant difference in the α-diversity and the microbial community composition between Control and CRS (Guo et al., 2019; Yang et al., 2021). Therefore, the results of the impact of CRS on microbial diversity are not consistent, which may be related to the modeling schedule and grouping design. Interestingly, Jiang et al. reported that CRS treatment for 3 weeks significantly inhibited the α-diversity (Chao1 and PD whole tree) of male rather than female mice (Jiang et al., 2023), indicating that gender is also one of the factors affecting microbial diversity. Indeed, females were specifically examined considering their greater stress vulnerability, HPA axis dysregulation, and higher predisposition to stress-induced anxiety compared with males (Heck and Handa, 2019). The present study did not consider the role of gender differences in CRS and the anxiety-like effect of EA, which should also be elucidated in the future. Notably, the changes in microbiota after CRS modeling (Control vs. CRS) were considerably more pronounced than those following sham EA intervention (Sham vs. CRS + fEA). This difference may be attributed to the recovery of the bacterial community 1 week after intervention, although the potential impact of anesthesia on the microbiota cannot be discounted (Serbanescu et al., 2019; Wang et al., 2022). In addition, fecal collection and behavioral testing were conducted after CRS, and the correlation between the two was analyzed. However, changes in fecal microbiota cannot timely reflect changes in gut microbiota. It may be more meaningful to observe the impact of regulating specific bacterial populations through methods such as fecal microbiota transplantation on behavior.

Furthermore, our analysis revealed that *Lactobacillus_gasseri* was more abundant in the Sham group and it was positively correlated with the number of entries into open arms. Conversely, *Bacteroides_uniformis* was enriched in the CRS + fEA group and negatively correlated with the number of entries into open arms and the time the subjects remained in the center. *Lactobacillus_gasseri* is a probiotic (Selle and Klaenhammer, 2013) whereas colonization of *Bacteroides_uniformis* has been associated with the adverse effects of a depressive microbiome on behavior (Zhang et al., 2022). These findings suggest that the CRS intervention continues to impact the microbiota 1 week later. Importantly, *Eubacterium_coprostanoligenes* is a species that has a lipolytic function (Li et al., 1998). Its abundance exhibited a negative correlation with the time spent in the center, indicating that the anti-anxiety effects of EA may be linked to the regulation of lipid metabolism.

## 5. Conclusion

In summary, our findings highlight that CRS leads to pronounced anxiety-like behaviors and disturbances in gut microbiota composition, and these effects can be partially mitigated through EA treatment. We further investigated the specific microbial species associated with anxiety-like behaviors at the species level, identifying 13 species linked to the anxiety-like responses induced by CRS. However, it remains to be explored how longer-duration and varying parameters of EA may impact gut microbiota composition.

## Data availability statement

The original contributions presented in the study are included in the article/supplementary material, further inquiries can be directed to the corresponding author.

## Ethics statement

The animal study was approved by the research procedures conducted in this study received approval from the Ethics Committee of Xi’an Gaoxin Hospital under the reference number 2023-GXKY-0011. The study was conducted in accordance with the local legislation and institutional requirements.

## Author contributions

JB: Conceptualization, Investigation, Writing – original draft. J-QW: Conceptualization, Data curation, Writing – original draft. QT: Investigation, Writing – original draft. FX: Formal analysis, Funding acquisition, Writing – original draft. WZ: Methodology, Writing – original draft. HH: Conceptualization, Writing – review & editing.

## Footnotes

^1^ http://drive5.com/uparse/
